# The Assessment of the Real-Time Radiative Properties and Productivity of *Limnospira platensis* in Tubular Photobioreactors

**DOI:** 10.3390/life12071014

**Published:** 2022-07-08

**Authors:** Manuel Vicente Ibañez, Rodrigo Jorge Leonardi, Felix Krujatz, Josué Miguel Heinrich

**Affiliations:** 1 Facultad de Bioquímica y Ciencias Biológicas (FBCB), Universidad Nacional del Litoral (UNL), Ciudad Universitaria (Paraje El Pozo), Santa Fe 3000, Argentina; rleonardi@fbcb.unl.edu.ar (R.J.L.); j.heinrich@fbcb.unl.edu.ar (J.M.H.); 2 Institute of Natural Materials Technology, TU Dresden, Bergstraße 120, 01069 Dresden, Germany; felix.krujatz@tu-dresden.de; 3 Biotopa gGmbH—Center for Applied Aquaculture & Bioeconomy, Bautzner Landstraße 45, 01454 Radeberg, Germany; 4 Faculty of Natural and Environmental Sciences, University of Applied Sciences Zittau/Görlitz, 02763 Zittau, Germany

**Keywords:** light availability, photobioreactor, cyanobacteria, radiative properties, growth kinetics

## Abstract

The development of tools to predict the photobioreactors’ (PBRs) productivity is a significant concern in biotechnology. To this end, it is required to know the light availability inside the cultivation unit and combine this information with a suitable kinetic expression that links the distribution of radiant energy with the cell growth rate. In a previous study, we presented and validated a methodology for assessing the radiative properties necessary to address the light distribution inside a PBR for varying illuminating conditions through the cultivation process of a phototrophic microorganism. Here, we sought to utilise this information to construct a predictive tool to estimate the productivity of an autotrophic bioprocess carried out in a 100 [L] tubular photobioreactor (TPBR). Firstly, the time-dependent optical properties over ten batch cultures of *L. platensis* were calculated. Secondly, the local volumetric rate of photon absorption was assessed based on a physical model of the interaction of the radiant energy with the suspended biomass, together with a Monte Carlo simulation algorithm. Lastly, a kinetic expression valid for low illumination conditions has been utilised to reproduce all the cultures’ experimentally obtained dry weight biomass concentration values. Taken together, time-dependent radiative properties and the kinetic model produced a valuable tool for the study and scaling up of TPBRs.

## 1. Introduction

The ability of eukaryotic microalgae and cyanobacteria to convert light and carbon dioxide into chemical energy has attracted biotechnologists as well as various companies for the last six decades [1]. Besides the possibility of cultivating microalgae in arid areas, flood plains or lands not fit for agriculture, these organisms can produce a wide variety of products, from high-value proteins, pigments or fatty acids to energy-rich lipids [2]. Among these, the cyanobacterium *Limnospira platensis* [3,4] is considered a safe (GRAS) species and a natural producer of vitamin B12, antioxidants and proteins [5]. Its biomass is widely used as a health superfood, feed supplement and source of fine chemicals, representing a worldwide annual production of around 10,000 tons [6].

Concerning the cultivation of photoautotrophic microorganisms, abundant work has been published that considers different lighting conditions, alternative geometries for the PBRs and the impact of low and high values of the biomass concentration on the performance of PBRs [7,8,9,10]. While open PBRs, such as ponds and raceways, are economically efficient choices for the mass production of low-value biomass or processes such as wastewater depuration, high-value applications typically demand a level of quality, control and homogeneity that closed systems can only meet [2,11]. Among these, TPBRs are an established technology that gained popularity in photobiotechnology over the last two decades [12]. These units are composed of two different parts: on the one hand, the place on which the radiant energy collection takes place, namely the solar loop, and on the other hand, a degasser, on which mass transfer processes take place, especially O_2_ desorption. This spatial differentiation of light-harvesting and gas exchange processes allows the optimisation of the photosynthetic performance of the PBR via modifying some parameters such as the diameter and length of the tubes or the linear velocity of the liquid. Although the latter and the easiness of scalability of a TPBR make them more attractive than other PBR types, there are some drawbacks related to high economic costs concerning their installation, biofilm formation and sufficient oxygen removal to avoid photooxidative stress [10].

**Figure 1:**
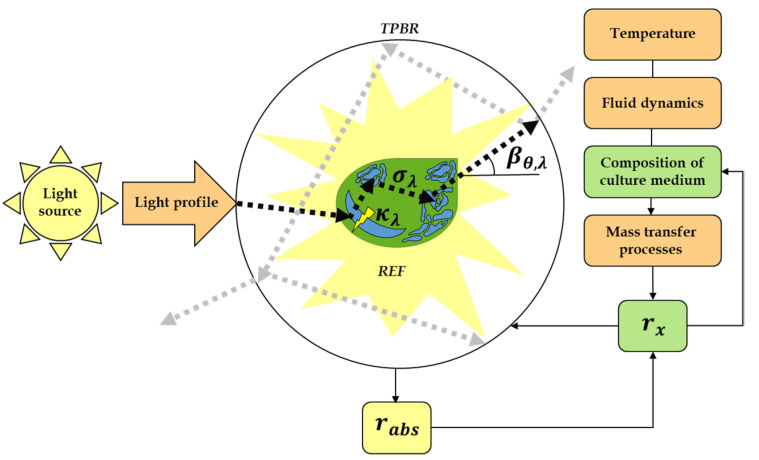
The summary of the interaction of the significant variables that affect a TPBR performance and the ones that allow the calculation of the REF. Any TPBR can be understood by treating the three major components of the system separately: the light (yellow), the geometry of the system and operating conditions (orange) and every aspect concerning the biology of the cultured microorganism (green). Adapted and reprinted from [20] with permission.

In the last few years, the knowledge regarding the REF in microalgae cultures, including light scattering and absorption, has expanded rapidly, as light is the most crucial factor driving photosynthesis [10]. It is recognised that algae growth rate and biomass content depend on light availability and spectral composition [13]. At the same time, during the progression of a batch run, the REF within it, and the composition of the liquid medium underlies a significant dynamic change [14,15]. Consequently, these variations trigger different adaptative processes such as adjusting strategies for capturing and dissipating radiative energy or the modification of cellular physiology and cell cycle control [16]. Thus, the performance of the culture results from considering these environmental conditions throughout the assessment of the phototrophic suspension radiative properties [17,18] (Figure 1).

In a previous investigation, we presented a model of well-mixed microalgal suspensions, considering them as a continuum with absorption and scattering centres homogeneously dispersed within the PBR volume [7]. Given the temperatures involved in microalgal cultures, light could be assimilated as a gas of photons which move in different directions with the speed of light. Based on this physical model, Monte Carlo algorithms were developed and computationally implemented with different purposes: one for the determination of the optical properties (OPs) of the suspensions of photosynthetic microorganisms (the spectral absorption ($\kappa_{\lambda }$) and scattering ($\sigma_{\lambda }$) coefficients, and the scattering phase function ($\beta_{\theta ,\lambda }$)) [19,20] and the other for the simulation of the REF in PBRs [8,21,22]. As a result, it was possible to predict the value of the local volumetric absorption rate of photons ($r_{\lambda }^{abs}(\underline{r},t)$) and its change with the microalgal culture growth, including the changing degree of stratification of light in the reactor [8,23].

Recently, we presented and validated a methodical perspective about the reproduction of outdoor lighting conditions at the laboratory scale and how this information may be employed for scaling up PBRs [10,24]. Later on, we published and validated a methodology for calculating the optical properties necessary to address the light distribution inside a TPBR for changing illuminating conditions through the cultivation process of an eukaryotic microalga [20]. Here, we sought to utilise this information to construct a predictive tool to estimate the autotrophic bioprocess productivity in a TPBR of 100 litres for a commercially well-known cyanobacterial strain, considering the geometry of the system, the nature of the lighting system in quantity and quality and the physiological state of the cyanobacterial cells measured as the radiative properties of the autotrophic suspension. 

Lastly, this preliminary study provides information about the kinetic behaviour of *L. platensis* under industrially relevant culturing conditions for ten cultivations, solely calculated from an energy balance. The latter highlights the possibility of considering the time-dependent radiative properties of autotrophic cultures as a design variable. Some authors have pointed out the necessity of considering time-dependent radiative properties for improving the predictions about the PBR production performance [10,25]. Even though our perspective holds the potential of allowing the design of cultivation strategies that considers adjusting light quantity or quality in response to the monitoring of the OPs, the present study was not meant to optimise the PBR under study. Proper optimisation is possible, but firstly the OPs of the unit under analysis should be addressed to commence pointing in this direction. Inasmuch as we faithfully continue reproducing the behaviour of eukaryotic algae and cyanobacteria in this way, more accessible ways of optimising PBRs based on quantifying the microalgal optical properties as a design, control or operating variable may take place.

## 2. Materials and Methods

### 2.1. Strain and Cultivation Conditions

The batch cultivations of *L. platensis* PCC7345 (PCC, Paris, Frence) were performed in a 100 [L] TPBR of MINT Engineering GmbH (Dresden, Germany). This TPBR is mainly composed of two units: the solar loop in which biomass growth occurs and the degassing tank. The PBR has 19 plastic tubes with an inner diameter of 0.0606 [m] and a total tube length of approximately 33 [m]. The liquid circulates with a pumping rate of 0.35 [m s^−1^] (15 [W]) and air gassing rates of 0.03 [vvm]. Additionally, oxygen is removed from the suspension by introducing air to the liquid in the degassing tank. The gas insertion is supposed to be accomplished by the outflow of the solar loop encountering the liquid surface in the degassing tank and the resulting mixing movements (Figure S2). In the exponential growth phase, a concentrated inoculum was prepared in an illuminated chamber to start the cultivation with a biomass concentration equal to 0.1 [g L^−1^]. To the end of cultivating cyanobacterial cells under industrially relevant physiological conditions, all the cultivations were carried out in a simplified Spirulina medium, including an inorganic plant fertiliser as the source of macro- and micro-nutrients (dosed as 0.5 mL of liquid fertiliser per litre of growth medium). The approximated composition of these reduced growth medium is: NaHCO_3_—8.0 [g L^−1^], NaNO_3_—0.21 [g L^−1^], NaCl—1.0 [g L^−1^], KNH_4_—1.85 × 10^−2^ [g L^−1^], KH_2_PO_4_—1.5 × 10^−2^ [g L^−1^], K_2_O—3 × 10^−2^ [g L^−1^] and traces of boron, copper, iron, molybdenum and zinc.

The reactor was unilaterally illuminated with warm-enriched light by four pairs of facing LED tubes (18 [W]) that were vertically mounted in the interspace of the solar loop. The whole lighting system (Figure S4) was fully characterised in its spectral (Figure S1a) and directional composition (Figure S1b). The total length of each LED lamp is 1.2 [m] and the inner diameter is 0.26 [mm]. Each lamp provides a total photosynthetic photon flux (PPF) equal to 30.08 ± 0.12 [µmol s^−1^], making a total PPF of 240.64 [µmol s^−1^] that is emitted in the direction of the PBR tubes. The latter PPF was assessed by making an energy balance through a MC Matlab routine. The values of the photosynthetic photon flux density (PPFD) at different distances from the light source were recorded employing a DK-PHAR 2.010BS PAR-quantum sensor (deka Sensor + Technologie Entwicklungs- und Vertriebsgesellschaft GmbH, Teltow, Germany). The distance from each LED lamp to the nearest point of the PBR walls is 60 [mm]. Lastly, Figure S5 shows the distribution of the photosynthetic photon flux density (PPFD) over the surface that faces the central tube of the TPBR.

### 2.2. Cell Dry Weight Concentration (C_x_) and Optical Density (OD750)

Culture samples of *L. platensis* PCC7345 were analysed using a UV/VIS spectrophotometer (GENESYS 150, Thermo Fisher, Waltham, MA, USA). The optical density (OD_750_) was measured at 750 [nm] and was calibrated against *C_x_* [g L^−1^] of *L. platensis* PCC7345 using the following correlation (in triplicate, [26]):

(1) $$C_{x}^{}=0.86\;\times \;OD_{750}$$

Aiming to assess *C_x_*, 15 mL of *L. platensis* PCC7345 suspension was passed through a glass microfiber filter (VWR International, Delaware Valley, PA, USA, mesh size: 1 [µm], in triplicate) and was then washed three times using 15 mL deionised water and dried for 24 h at 103 °C (Memmert GmbH + Co.KG, Schwabach, Germany). Finally, the weight difference of dried biomass was determined to calculate *C_x_* [26].

### 2.3. Assessment of the Radiative Properties (OPs)

The optical properties corresponding to each sample ($\kappa_{\lambda }$, $\sigma_{\lambda }$, $\beta_{\theta ,\lambda }$) were evaluated following the methodology developed and described by Ibañez et al. [20]. The simulation algorithm was built on the MC stochastic method. The methodology implies utilising a custom-designed system composed of a light source, a detector, two optical fibres connected to both devices and the other edge of the fibres into a plastic 3D-printed device and a custom-made device cuvette filled with 2 [mL] sample volume. The source of light utilised in this work is a tungsten halogen HL-2000 lamp (Ocean Insight, Ostfildern, Germany). The lamp’s stable wavelength range is 360–2400 [nm], and it maximises light throughput with adjustable focus and alignment through an SMA 905 connector (Ocean Insight, Ostfildern, Germany) that provides accuracy to the light collection of optical fibres. The detector employed was a Red Tide 650 spectrometer (Ocean Insight, Ostfildern, Germany). The spectrometer’s resolution is 2 [nm] with a detectable spectrum ranging from 350 to 1000 [nm]. The spectral data have been obtained employing SpectraSuite (Ocean Insight, Ostfildern, Germany). The latter was configured with the lowest integration time and default options disabled. The optical fibres used were two pieces of stainless steel QP400-2-UV-BX fibres (Ocean Insight, Ostfildern, Germany), with a robust transmission capacity from 300–1100 [nm] and 400 [µm] diameter size.

The ad hoc simulation algorithm was written and executed in MATLAB (2020a). The computational flow diagram, including the decision nodes of the stochastic algorithm, can be found in Ibañez et al. [20]. The solver employed in all the regressions was *lsqcurvefit + multistart*. The latter is a non-linear least-squares solver that finds the coefficients necessary to minimise the difference between observed and input data. Under a default step tolerance and function tolerance, every independent data series was adjusted utilising the trust-region-reflective algorithm. Finally, the non-linear 90% confidence intervals of any calculated parameter of interest were calculated through the *nlparci* solver of MATLAB. This last required the Jacobian matrix of each experimental data series regression under analysis.

### 2.4. Modelling, Simulation and Analysis of Radiant Energy Field (REF)

The physical and mathematical model upon which the simulation of the REF in the culturing medium relied was carried out following the methodology developed and described by Heinrich et al. [7,19]. The simulation algorithm was based on the MC stochastic method. A probability was assigned to each possible outcome of the events that photons can undergo as they reach deeper regions in the culturing medium. Along the way from the light source to any point in the cyanobacterial suspension, their intrinsic trajectories may be deflected by scattering effects due to elastic interactions between photons and suspended cells or even reach an abrupt end due to the local absorption of the tracked photon. On sound physical grounds, a probability of occurrence is assigned to these events through expressions that include both the spectral absorption coefficient ($\kappa_{\lambda }$) and the scattering coefficient ($\sigma_{\lambda }$). The probability of occurrence of scattering is used together with the phase function ($\beta_{\theta ,\lambda }$), which enables the choice of a new direction of photon propagation in the case of photon scattering [20]. This way, the trajectory of each photon is described on a probabilistic basis until it is absorbed or leaves the culture through the boundaries of the PBR. Additionally, in the present study, the trajectory of each light beam may lead them to pass through the space between the tubes of the TPBR and be lost or have the chance to impact any of the 19 tubes composing the geometry of this culture system. Therefore, each tube and connector were treated independently.

The computational steps diagram, including the MC algorithm’s decision nodes, was presented in Leonardi et al. [22]. The ad hoc simulation algorithm was designed and run in MATLAB (2020a). Concerning the REF properties, the definitions and units recommended in Alfano et al. [27] were employed. Lastly, the regression of kinetic parameters was carried out using *globalsearch + fmincon* solver. Here, the error function was established as the non-linear squares minimising the sum of the differences between the experimental and model-predicted values. Every independent data series was adjusted utilising the trust-region-reflective algorithm under a default step tolerance and function tolerance. Finally, the model’s performance and the consequent calculation of the associated errors were performed utilising *fitnml* from the MATLAB Statistics and Machine Learning Toolbox.

## 3. Results and Discussion

In a purely phototrophic culture, microalgae are dependent on absorbing light energy to meet their demand for cellular functions and growth. Massive culture of cyanobacterial biomass in industrial PBRs requires a high biomass concentration and optically dense cultures capable of absorbing a high proportion of the collected light through its boundaries. Therefore, in PBRs, intense light gradients may be present naturally, especially in outdoor units. Not all the photons are equally absorbed in the suspension. Not all reactor zones are equally productive regarding photosynthetic growth because their productivity depends on the local photon availability and their wavelength. This last may lead to the coexistence of oversaturating and unlit zones, a phenomenon that is widely known as *self-shading*, giving place to the generation of simultaneous oversaturating, or further, photoinhibition zones and respiration zones, which could cause a noticeable loss of photosynthetic efficiency and a significant decrease in the TPBR productivity if not managed properly. 

The local volumetric spectral rate of photon absorption $r_{\lambda }^{abs}(\underline{r},t)$ is the number of photons of wavelength λ locally absorbed per unit time and unit volume of culture for a particular moment throughout the length of a cultivation process. Accessing $r_{\lambda }^{abs}(\underline{r},t)$ at every location in the culturing medium is key to understanding the factors affecting light absorption by microalgae for a given reactor setup and comparing the performance of different reactor configurations operating under different conditions concerning this process.

The present work presents an approach to measure the optical properties of microalgae suspensions based on the radiation simulation employing an MC method and the employment of these in the evaluation of the light field inside a TPBR. The latter’s advantage is that it allows the handling of complex reflexive systems and the optical phenomena occurring within industrial-scale TPBRs without introducing simplifications to solve complicated mathematical approaches. Under this perspective, the culture is a continuum and the cells are centres of absorption and scattering, with associated probabilities accounting for these events. After that, through an optimisation program, the experimental culture data will be used to determine ($\kappa_{\lambda }$, $\sigma_{\lambda }$, $\beta_{\theta ,\lambda }$) based on the assumption that detectable changes in light passing through the culture are faster in comparison with the biological processes involved in the progressive changes regarding the OPs. It is essential to note that this way of assessing the REF is independent of pigments and biomass concentrations. The latter is not a minor statement, as a model of interaction linking light and the culture must be applied to do so, and due to the system’s complexity, there is a lack of agreement between the different empirical approaches. Still, there are no studies where the inner structures and the cell’s shape are included in the model when describing cyanobacterial cells with complex shapes.

Lastly, as this methodology allows more intuitive ways of calculating the OPs of phototrophic microorganisms in the complex context of the evolution through the time of the REF inside a TPBR, a suitable kinetic expression was chosen to link the properties of the light field with cell growth. The accuracy and extension of this approach are analysed in the present section.

### 3.1. Absorption, Scattering Coefficients and the Scattering Phase Function of L. platensis

The cyanobacterium *Limnospira platensis* [3,4], often referred to as Spirulina for commercial purposes, forms multicellular, filamentous structures known as coiled trichomes [28]. The cylindrical cells with diameters of 6–12 [µm] are arranged in helices with diameters ranging from 30 to 70 [µm] that typically possess a length of around five to seven coils. The ultrastructure of an *L. platensis* cell exhibits a similar cell organisation to that of a typical prokaryotic cell with a Gram-negative cell wall. Nevertheless, *L. platensis* consists of thylakoids formed by membrane systems arranged in bundles parallel to the longitudinal cell wall. This cyanobacterium may conduct oxygenic photosynthetic processes due to the thylakoid membrane-integrated photosystem II (PSII), which is, among others, composed of chlorophyll *a*, and the presence of phycobilisomes (PBS) [29]. PBS are supramolecular light-harvesting complexes composed widely of phycobiliproteins, such as phycocyanin or allophycocyanin, having the presence of other polypeptides. Phycobiliproteins exhibit colouring chromophores, the so-called phycobilins attached by covalent bindings. Thus, the bluish colour of *L. platensis* is caused by its primary composing pigment, phycocyanin, among others. Moreover, carboxysomes that possess polyhedral inclusion bodies can be found in central cytoplasmic regions of *L. platensis*. These micro-compartments of a crystal-like structure store the enzyme ribulose-1,5-bisphosphate carboxylase/oxygenase (RuBisCO) [28].

In *L. platensis*, depending on the culture conditions, it was found that the size of the cells can vary in response to low or high PAR lighting conditions, whether the lighting conditions change in quality and quantity or there are organic carbon sources present in the liquid [29]. Additionally, a morphological alteration is regulated by light to the transcriptional level [30]. At the beginning of a cultivation process, a photoacclimation effect is related to the loosening process of *L. platensis* spirals, which enables the cells to receive more light for photosynthesis. On the contrary, a tightening process also allows the cells to shade themselves when solar radiation becomes excessive [29]. The quantity and size of PSII can also change in response to the environment in this cyanobacterium and the composition of the accessory pigments [31]. Chlorophyll *a* levels were reported to be altered in response to different illuminating conditions, as well as beta-carotene and phycobiliproteins [32]. Furthermore, the architecture of PBSs changes drastically due to high radiation in the PAR spectral range [32].

**Figure 2:**
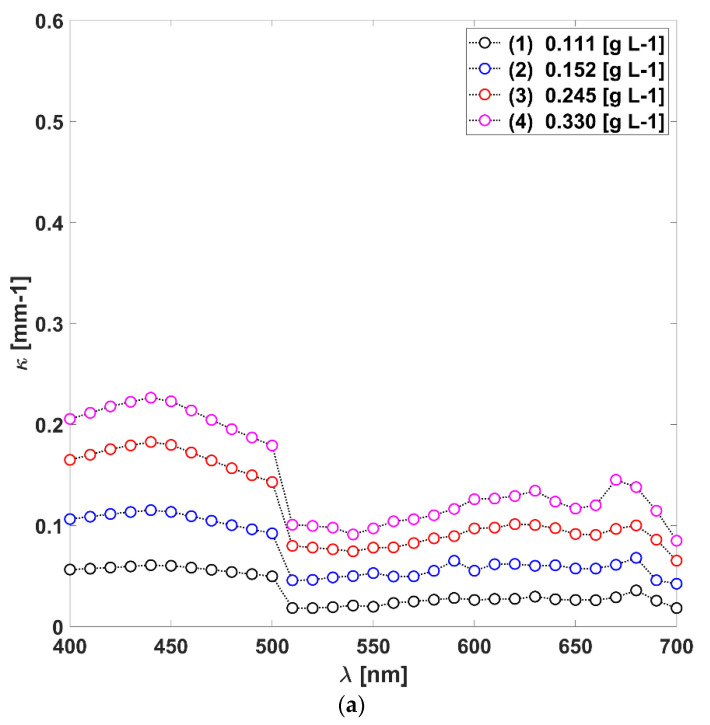

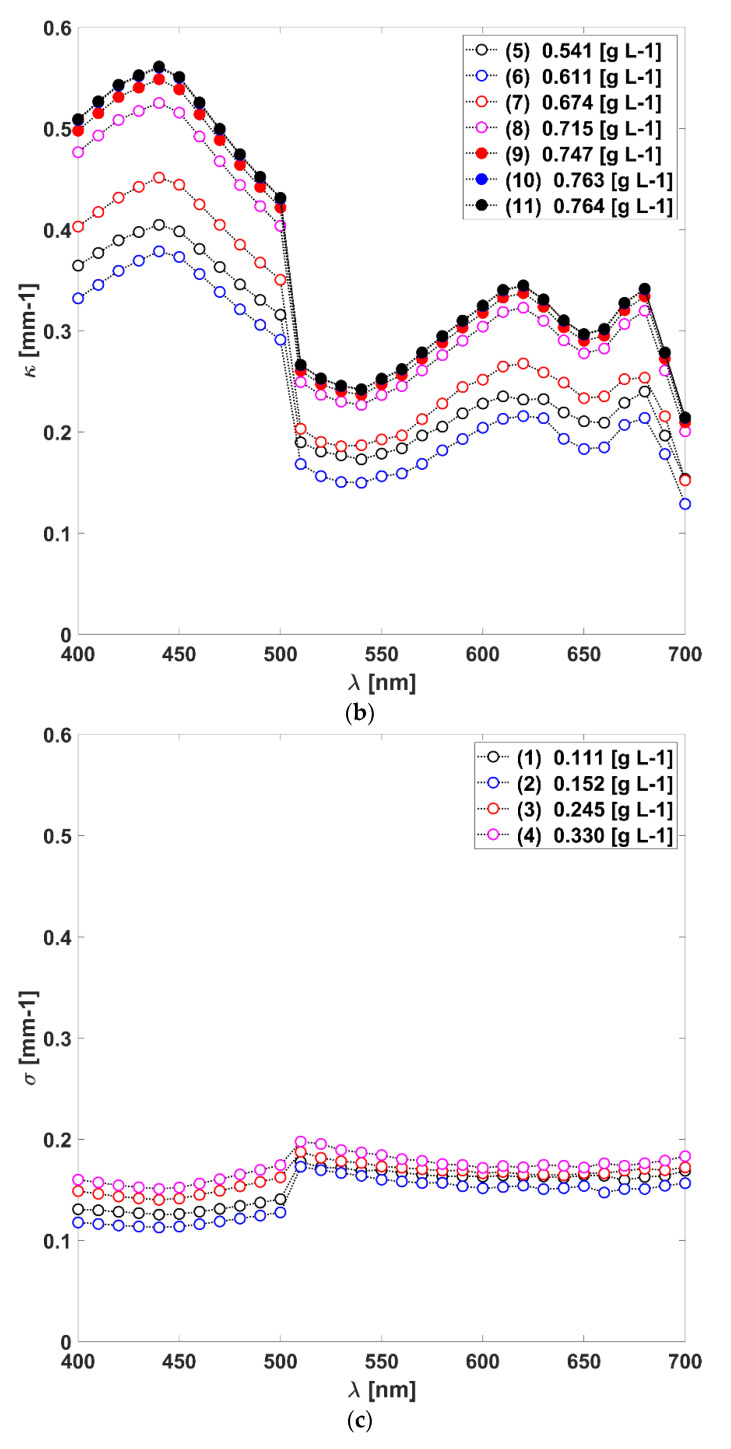

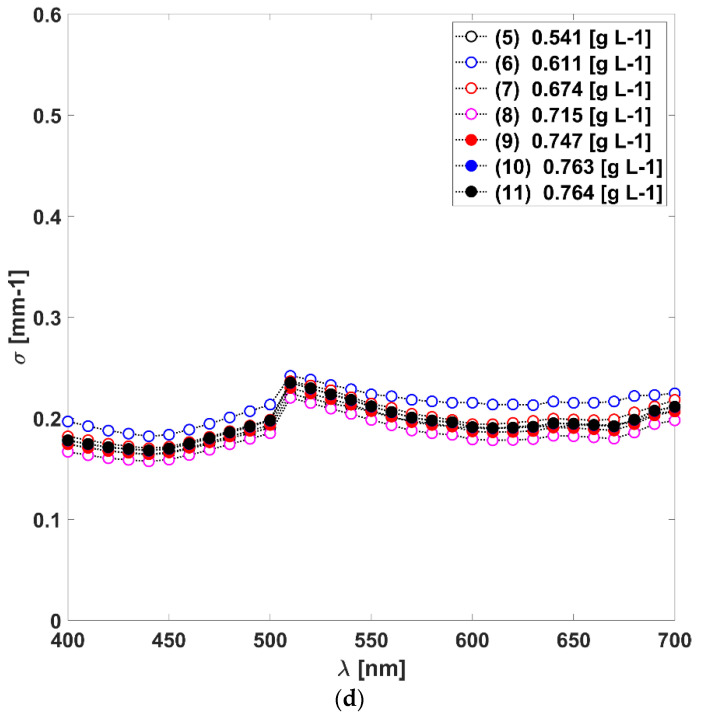
(**a**,**b**). The absorption $\kappa_{\lambda }$ and scattering coefficients of eleven samples. (**c**,**d**). The spectral scattering $\sigma_{\lambda }$ coefficients of eleven samples.

Here, we sought to investigate the radiative properties of a phototrophic culture of *L. platensis* based on an energy balance. The latter allows the construction of a continuum medium of radiation properties in the culture volume in a single period, although unevenly distributed in space and wavelength. Then, the OPs related to the REF at this moment, fated to every chemical or biological alteration in the system, such as the ones mentioned in previous paragraphs, were calculated. Figure 2a,d present ten sets of $\kappa_{\lambda }$ and $\sigma_{\lambda }$ coefficients in the PAR spectral range. The trend in the coefficients’ variations in the wavelengths region from 400 to 700 [nm] has shown expectable results in comparison with other members of Cyanophyceae [33].

As for absorption, there are larger values at those wavelengths where the chlorophyll pigments are active to light. Free Chl *a* absorb around 435 and 676 [nm]. In Figure 2a,b, these peaks are smoother and present shifts due to two effects: firstly, Chl molecules are supported by other proteins in the core antennas of the LHC, which creates an overlapping in the spectrum of these free substances; secondly, as it was mentioned before, it is natural in *L. platensis* and in the synthesis of carotenoids [34]. Lutein and zeaxanthin, as representatives of all the different α- and β-carotene intermediates, have absorption maxima around 445 and 474 [nm] and 480 [nm] on an individual basis [11]. The latter also contributes to the spectral peaks overlapping the PAR spectral range, explaining the other wavelength variations ranging from 550 to 650 [nm]. Lastly, the PBSs that take shape in antenna-like arrangements absorb light in the wavelength range of around 540 to 650 [nm], as they capture sections of the solar spectrum not used by chlorophyll molecules and very efficiently transmit energy to the photosystems where charge separation takes place [31]. It is notable to see how the peaks related to phycobiliproteins become more important towards the increasing biomass content relative to the peaks of Chl *a* (Figure S3).

Regarding the fate of the non-absorbed light, when absorption occurs in the LHC and the amplitude of an electromagnetic field changes, its phase will change accordingly, producing inelastic scattering. From Figure 2c,d, the habitual trending in the light dispersion’s behaviour for an autotrophic suspension can be observed. Light scattering tends to be higher than microalgae absorption efficiency [35]. Even though the $\sigma_{\lambda }$ depends on wavelength as the $\kappa_{\lambda }$, this wavelength selectivity is not that sharp for the cyanobacterium scattering spectrum [36]. The latter may be attributed to the dominant influence of the non-absorbing cell components over the $\sigma_{\lambda }$, although pigments exert an effect through fluorescence and selective absorption [15]. The ensuing $\sigma_{\lambda }$ to the spectral range 500–560 [nm] are the highest values (Figure 2c,d), corresponding to the lowest $\kappa_{\lambda }$ in Figure 2a,b.

Last but not least, the ${\left({\bar{\mu }}_{n}^{*}\right)}_{PAR}$ values composing the most favourable angles represent the forward scattering pattern of *L. platensis* and $\beta_{\theta ,\lambda }$ are listed in (Table S1). As it has been observed for *C. zofingiensis* [20], there are variations in the calculated values of ${\left({\bar{\mu }}_{n}^{*}\right)}_{PAR}$. Here, rather than elucidate the specific influence of a single element, the nature of these variations is generally related to all of the complex non-absorbing components that may be present within the cells. So far, the understanding of the individual organelle–light interaction is not sufficiently wide to present a $\beta_{\theta ,\lambda }$ function more than partially biased by wavelength. Therefore, the values of $\beta_{\theta ,\lambda }$ will be utilised as the averaged values across the entire PAR spectral range.

### 3.2. Modelling and Analysis of Radiant Energy Field within the TPBR

The optimisation and control of light transfer in PBRs on which an autotrophic process is carried out are bound to the close relationship between the radiation source’s emission characteristics, the reactor’s geometry and the suspension’s OPs that reside within it. Combining these three components in the radiative transfer equation (RTE) allows access to know the light availability inside the unit, which accounts for the physiological state of the culture at a given time. Cells modify the light field and light affects the life of the cells. Light exposure and nutrient-level alterations trigger a set of physiological processes in microalgae on both transcriptional and metabolic levels. These processes affect the OPs of the cells. Therefore, radiation characteristics and the size of the cells are not constant but continually changing as a response to variations in the spectral density distribution of photons $e_{\lambda }^{}(r,t)$, among other factors [15].

If $\kappa_{\lambda }$, $\sigma_{\lambda }$ and $\beta_{\theta ,\lambda }$ were calculated experimentally, $e_{\lambda }^{}(\underline{r},t)$ can be addressed as follows:

(2) $$e_{\lambda }^{}(\underline{r},t)=\int_{\underline{\overset{⌢}{\Omega }}}n_{\lambda }^{}(\underline{r},\underline{\overset{⌢}{\Omega }},t)\;d\underline{\overset{⌢}{\Omega }}$$

In Equation (2), $n_{\lambda }^{}(\underline{r},\underline{\overset{⌢}{\Omega }},t)$ is the local density number of photons with wavelength λ with a trajectory through the position $\underline{r}$ in the direction $\underline{\overset{⌢}{\Omega }}$, for a particular time t in the cultivation process. Thus, in agreement with the radiation transfer theory, $r_{\lambda }^{abs}(\underline{r},t)$ can be readily obtained through the use of the light speed constant and the spectral absorption coefficients:

(3) $$r_{\lambda }^{abs}(\underline{r},t)=c\;\kappa_{\lambda }(t)\;e_{\lambda }^{}(\underline{r},t)$$

**Figure 3:**
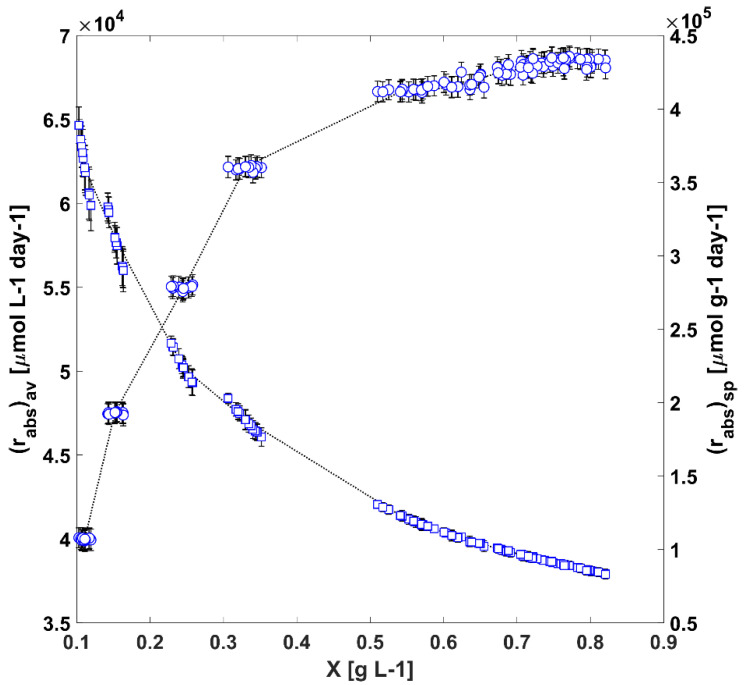
The evolution of the average (${\bar{r}}_{PAR}^{abs}$, ○) and specific average (${\bar{r}}_{PAR}^{abs,\;SP}$, □) rate of PAR photon absorption in the TPBR.

In Figure 3, it is possible to see the outcome of the numerical simulation of ${\bar{r}}_{PAR}^{abs}(t)$ and ${\bar{r}}_{PAR}^{abs,\;SP}(t)$, which is the result of summing up all the contributions within the photosynthetically active wavelength range for the total PBR positions, where:

(4) $${\bar{r}}_{PAR}^{abs}(t)=\frac{1}{V_{PBR}}\int_{0}^{\infty }\int_{400}^{700}r_{\lambda }^{abs}(\underline{r},t)\;g(r_{\lambda }^{abs},t)\;d\lambda \;dr_{\lambda }^{abs},$$

and

(5) $${\bar{r}}_{PAR}^{abs,\;SP}(t)=\frac{{\bar{r}}_{PAR}^{abs}(t)}{\bar{x}(t)}$$

In Equation (4), ${\bar{r}}_{PAR}^{abs}(t)$ is the average rate of PAR photon absorption in the TPBR, $V_{PBR}$ is the culture volume within the TPBR and $g(r_{\lambda }^{abs},t)$ is a measure of the frequency of occurrence of $r_{\lambda }^{abs}(\underline{r},t)$ values in the TPBR, or the volume distribution function in terms of the photon absorption rates, subject to the following normalisation condition:

(6) $$V_{PBR}=\int_{0}^{\infty }g(r_{\lambda }^{abs},t)dr_{\lambda }^{abs}$$

In Equation (5), ${\bar{r}}_{PAR}^{abs,\;SP}(t)$ is the average specific rate of PAR photon absorption in the TPBR, while $\bar{x}(t)$ is the cell biomass concentration for a given time alongside the cultivation process.

In the experimental cultivations carried out, the amount of radiation absorbed increases with the biomass concentration. However, when a larger number of cells captures light, the amount of light absorbed per biomass unit is reduced and vice versa (Figure 3). Even though the ${\bar{r}}_{PAR}^{abs}(t)$ values increased with the amount of suspended biomass, we should consider that as the $\kappa_{\lambda }^{}(t)$ coefficients evolved, the contribution of each $r_{\lambda }^{abs}(\underline{r},t)$ to the average rate value changed drastically.

**Figure 4:**
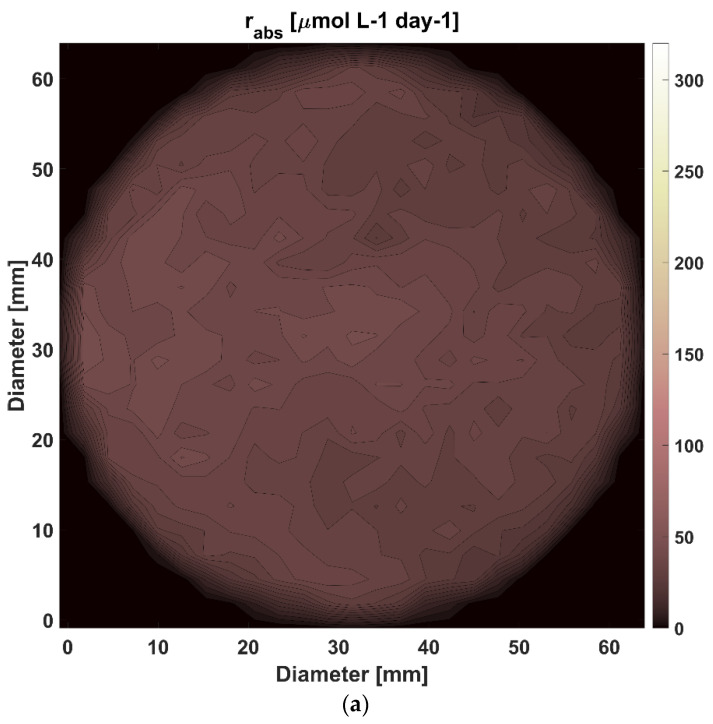

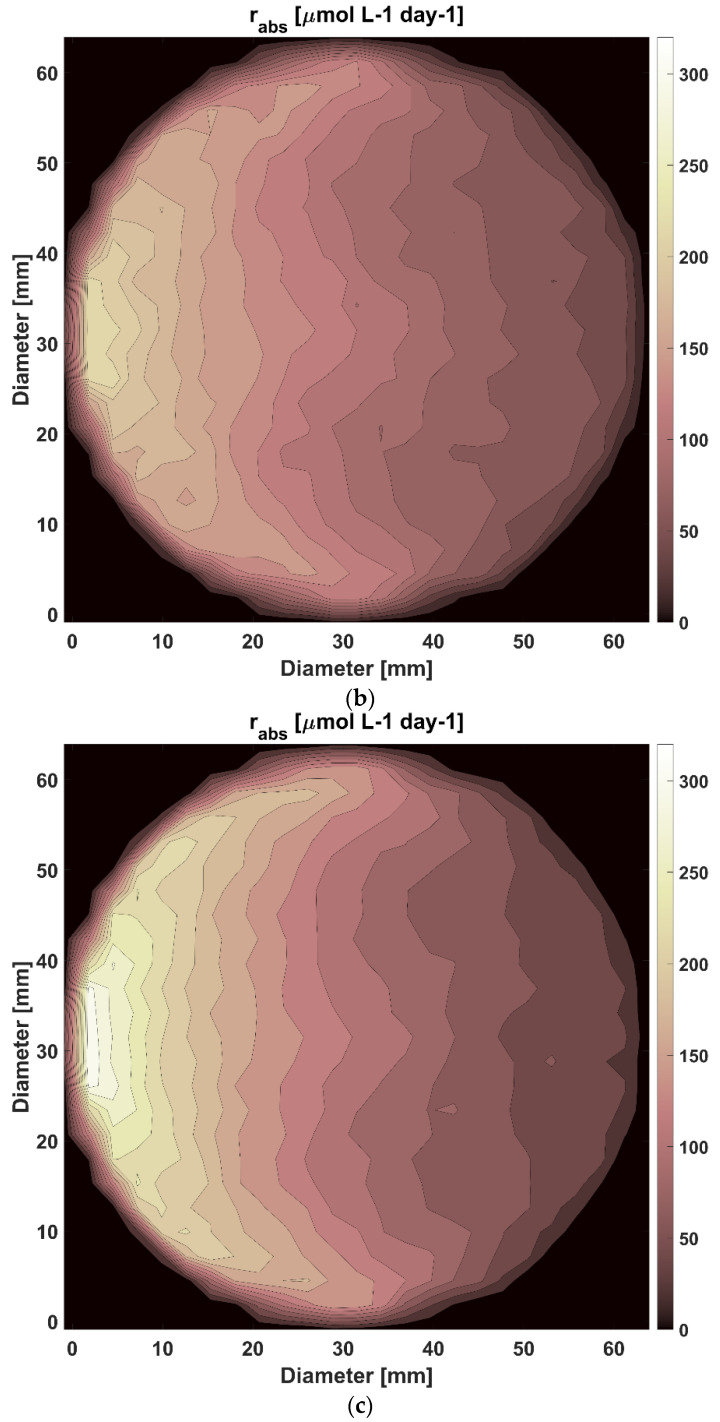
(**a**,**c**). $r_{PAR}^{abs}(\underline{r},t)$ distribution profiles as a function of the diameter of the central tube of the TPBR solar loop at a corresponding cross-section equal to the median length of the tube. The suspended biomass concentration for a, b and c particular moments alongside the cultivation process is 0.111 (**a**), 0.541 (**b**) and 0.764 (**c**) [g L^−1^], respectively.

Light is unevenly distributed in the reactor because of the phenomena of absorption, scattering and reflection associated with the transfer of radiant energy and with the configuration of the PBR. Figure 4a,c present how the stratification of light changes along with the cyanobacterial growth for a single tube of the 100L-TPBR, giving rise to zones with different local volumetric rates of absorption $r_{\lambda }^{abs}(\underline{r},t)$ and different spectral volumetric density $e_{\lambda }^{}(\underline{r},t)$ of photons within the PAR spectral range. In Figure 4a, the $r_{PAR}^{abs}(\underline{r},t)$ values are very low for all radial distances, irrespective of the biomass concentration. For high biomass concentrations (Figure 4c), a significant fraction of the photons that enter the suspension are absorbed in zones close to the irradiated boundary, and only a tiny fraction is left to be absorbed in more distant zones. Whether this evolution of the light stratification effect is advantageous or adverse to biomass performance will rely on whether the positive impact of an increase in the $r_{PAR}^{abs}(\underline{r},t)$ values is outdone by the negative effect of a decrease in the efficiency in the radiant energy utilisation.

Finally, in light of this analysis and concerning the light source, it is possible to propose using radiation sources of higher energy output to circumvent the stratification effect. Nonetheless, this is not an option without shortcomings because the increase in the availability of radiant energy in the zones of concentrated suspensions already exposed to high light intensity may cause the saturation of the photosynthetic systems or may even be harmful to them [29]. Because of the latter, it is tempting to strongly support the previous selection and employment of the radiation source already chosen for this illuminating system, as it emits photons in wavelengths corresponding to a low or middle value of the absorption coefficient (Figure S1a) and this helps to facilitate light penetration into the culture avoiding the generation of excessively irradiated areas or respiration zones.

### 3.3. The Autotrophic Growth in the TPBR and Regression of Intrinsic Kinetic Parameters

As far as $r_{\lambda }^{abs}(r,t)$ values are concerned, the biomass concentration is an essential operating variable, which can be manipulated to balance the relative importance of deeper zones into the suspension [25] with the contribution of zones near the irradiated boundary, which will be reflected in that the profiles of rates of photon absorption will be less steep and vary across the PAR spectral range. These differences in $r_{\lambda }^{abs}(\underline{r},t)$ values for different wavelengths could be an interesting parameter in PBR operation conditions and design. In the case of concentrated suspensions, photons whose wavelength corresponds to the higher values of the absorption coefficient are absorbed in the zones closest to the irradiated boundary. Consequently, the deepest zones in a homogeneous suspension are relatively poorly illuminated by the energy of high photosynthetic value. These regions, “ill-lit” than the rest in what valuable energy for photosynthesis is concerned, have a meagre rate of photon absorption in the wavelength ranges of interest for photosynthesis.

In order to gain a deeper insight into the cyanobacterial biomass light dependence when light availability fluctuates, it is necessary to follow the REF changes alongside the cultivation process and link the growth kinetics with a suitable property of the light field. In the 100L-TPBR under analysis, the $r_{\lambda }^{abs}(\underline{r},t)$ values are not uniform (Figure 4c). Nonetheless, from previous studies performed with the unit, there is an agreement that the culture circulates in a turbulent regime. Thus, it is generally assumed that fluids circulating in turbulent conditions through pipes are radially well mixed. In accordance with this notion, the fluid elements move from the centre to the surface of the tube lines several times per second. In addition, this unit possesses a degassing tank on which several volume elements perfectly mix with each other before starting a new run into the pipes. This evidence strongly supports the conclusion that the solar loop of the 100L-TPBR operates through a well-developed plug flow regime. Then, the typical mixing time inside each of the “plugs” is much smaller than the typical time of cyanobacterial growth. Under these conditions, the cyanobacterial cells frequently turn from lighted zones to dark zones (and vice versa) many times and also perform several runs alongside the tubes before cellular replication occurs. Therefore, it is tempting to assume an integrated scenario and that the kinetics of cell growth is driven by ${\bar{r}}_{PAR}^{abs}(t)$.

In this work, a kinetic expression based on a simplified pathway of the light-dependent step of photosynthesis and the inclusion of time-dependent radiative properties have been applied [21]:

(7) $${\bar{r}}_{X}(t)=K_{3}\left(\sqrt{1+K_{2}\;{\bar{r}}_{PAR}^{abs,\;SP}(t)}-1\right)$$

In Equation (7), ${\bar{r}}_{PAR}^{abs,\;SP}(t)$ has been previously defined in Equation (5), while $K_{2}$ and $K_{3}$ are kinetic constants related to the intracellular rate of ferredoxin formation and the rate of net photosynthesis, as the energy harvesting stage is the growth limiting step. ${\bar{r}}_{X}(t)$ is the average biomass growth rate. Furthermore, the mass balance proposed for this TPBR operated in batch mode was established and later solved as follows:

(8) $$\frac{d\;\bar{x}(t)}{d\;t}={\bar{r}}_{X}(t)$$

**Figure 5:**
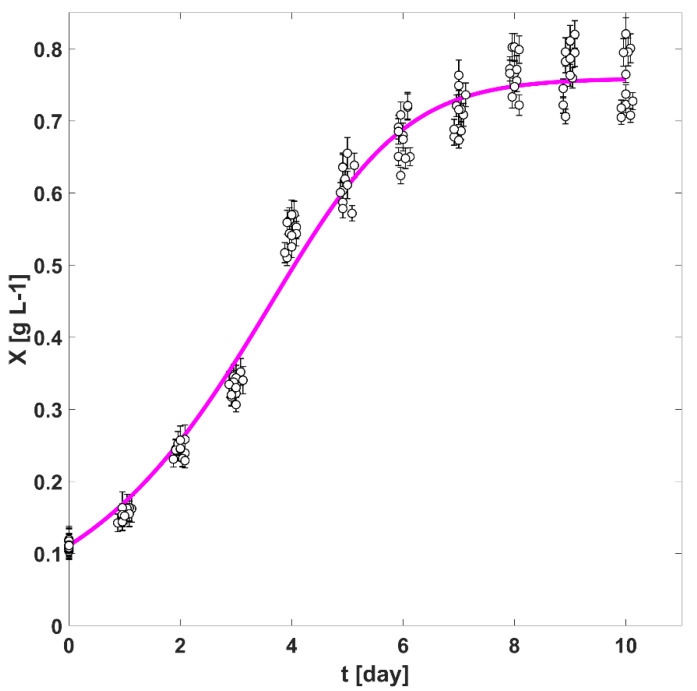
Experimental (○) and predicted (solid magenta line) biomass values for every one of the batch cultivation processes analysed in this study.

In Figure 5, the solid line represents the predicted biomass values for every one of the batch cultivation processes analysed in this study. The kinetic parameters related to physical quantities concerning the photosynthetic process are shown in Table 1. From Figure 5, it is possible to conclude that around the fourth day of cultivation for each one of the cultures, the rising biomass growth rate ${\bar{r}}_{X}(t)$ started to decrease. This corresponds to the situation shown in Figure 4b, in which is it pictured that the influence of ill-lit areas, far away from the irradiated boundaries of the tubes, starts to have a major contribution to the dispersion of the $r_{\lambda }^{abs}(\underline{r},t)$ around ${\bar{r}}_{PAR}^{abs}(t)$.

As the REF evolves, changes in the architecture and composition of the photosystems (PS), depending on the lighting conditions, have been observed in many species of cyanobacteria and green microalgae [10]. *L. platensis* [37] increased their Chl content per biomass unit weight when they were grown at low light intensities. Contrarily, some members of the green microalgae showed lower pigmentation content as light distribution in the TPBR became homogeneous [38]. Under low light intensity conditions, algae produce photosynthetic systems with greater capacity to capture photons. This increase in the photon uptake capacity is achieved by enhancing the synthesis of primary and accessory pigments [39]. In *L. platensis,* it has been observed that the ratio between the PSI and PSII is altered under the self-shading effect [30].

The light-to-biomass yield ($Y_{xp}^{}(t)$) depends on the proper kinetic coupling between the absorption of light, electron transport and carbon fixation processes [8].

**Figure 6:**
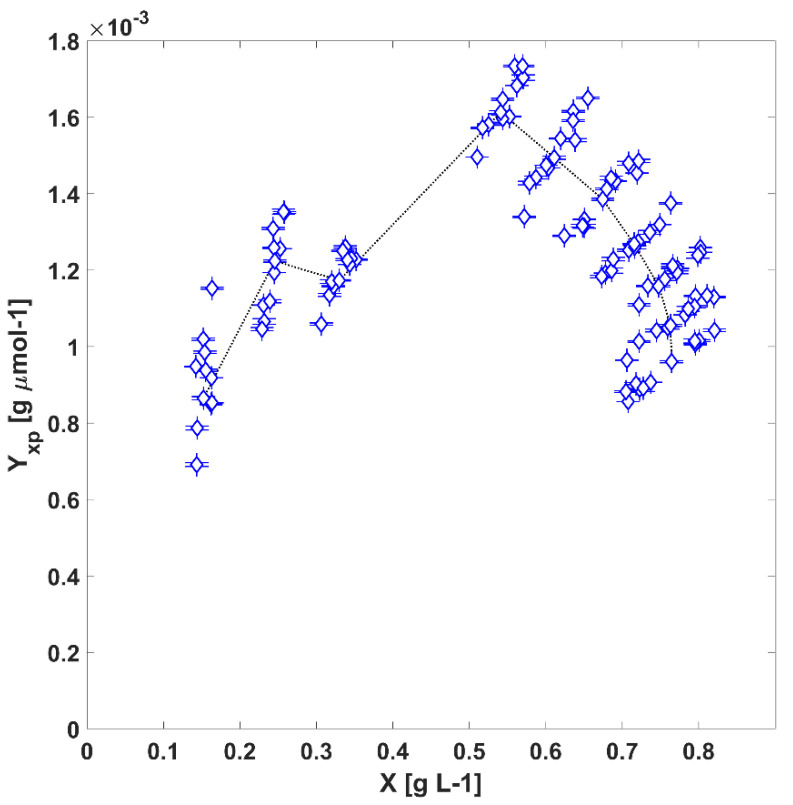
Light-to-biomass conversion values (◊) as a function of biomass concentration values studied in the TPBR.

In Figure 6, is possible to see the photosynthetic yield, $Y_{xp}^{}(t)$, where:

(9) $$Y_{xp}^{}(t)=\frac{{\bar{r}}_{X}(t)}{{\bar{r}}_{PAR}^{abs}(t)}$$

Here, an increase in biomass concentration brings about an increase in the amount of energy absorbed up to a certain biomass concentration value. Afterwards, the biomass productivity is reduced due to a lowering photoautotrophic growth efficiency. This reduction is related to the adaptive mechanisms discussed in previous paragraphs (Section 3.1). On the one hand, if the light absorption rate is greater than the speed at which electrons generated in the reaction centres are transported, a depletion in the available electron carriers takes place, and due to a lack of processing capacity, a more significant fraction of the absorbed energy is dissipated mainly as thermal energy to the adjacent volume acting as a thermal sink [22]. On the other hand, the efficiency in the use of photons is associated with the size of the complex light-capture antenna, which, as was highlighted in previous sections, in *L. platensis* suffers morphological changes regulated at the molecular level triggered by the naturally occurring stratification of light [23,30]. Even though there exists a clear dispersion over the biomass cultivation values that could be attributed to variations in other growth factors (such as varying temperatures [9] or nutrient starvation [40], oxygen mass transfer limitations and toxic effects of the oxygen evolution within the tubes [26,41] and poor mixing regime [25]), the predicted values show good agreement with the experimental values and the notion that light availability is the controlling factor driving the growth kinetics in the system under study. Lastly, the calculated TPBR mean areal productivity oscillates around 2.48 ± 0.06 [g m^−2^ day^−1^]. The latter value is up to six times lower than the reported areal productivities for similar PBRs [40], confirming the fact that the amount of light collected by the TPBR may be high, but the amount of energy supplied could be increased to improve the biomass productivity [41].

## 4. Conclusions

From a biotechnological point of view, the study of the light behaviour of photobioreactors is compulsory to find and employ a light exposure regime that provides the maximum conversion of light to biomass and high-value compound production. Unluckily, achieving high biomass growth rates and the best productivity of the desired target products is usually tricky. In this preliminary work, a kinetic expression for *L. platensis* growth under light controlling conditions has been applied based on an energy balance evaluated by assessing the cultivations’ time-dependent radiative properties. The ${\bar{r}}_{PAR}^{abs}$ values were calculated considering the photobioreactor’s uneven light distribution because of the absorption and scattering phenomena associated with the transfer of radiant energy and with the configuration of the PBR. Based on ${\bar{r}}_{PAR}^{abs}$, the growth of *L. platensis* as a function of time was simulated, showing good agreement with experimental data for similar cultivations carried out in the same unit and influenced by the same operating conditions. Even though the original focus of this work was not the optimisation of the TPBR, the possibility of assessing the suspension’s radiative properties through the cultivation time by this methodology highlights the option of designing experiences in which these coefficients may be taken as variables to optimise. Considering that a high degree of light stratification may affect *L. platensis*, measuring the optical properties could help adjust the light source’s quantity and quality to keep these parameters constant and regulate the light-to-biomass conversion values up to a maximum level.

## Figures and Tables

**Table 1:** Model parameters resulted by fitting the experimental average specific rate of PAR photon absorption in the TPBR and biomass concentration values.

Parameter	Value	Unit	Adjusted R^2^
$K_{2}$	$7.60\;\times \;10^{-12}\pm \;0.71\;\times \;10^{-12}$	$\left[\mathrm{day}\;g\;{\mu \mathrm{mol}}^{-1}\right]$	0.937
$K_{3}$	$2.66\;\times \;10^{4}\pm \;0.14\;\times \;10^{4}$	$\left[g\;L^{-1}\;{\mathrm{day}}^{-1}\right]$	

## Data Availability

Raw data of this research study are available by contacting the authors.

## Supplementary Materials

The following supporting information can be downloaded at: https://www.mdpi.com/article/10.3390/life12071014/s1.

## Abbreviations

$\kappa_{\lambda }$	absorption coefficient [mm^−1^]
$r_{\lambda }^{abs}$	local volumetric light absorption rate [µmol L^−1^ day^−1^]
$\sigma_{\lambda }$	scattering coefficient [mm^−1^]
$r_{x}^{}$	specific biomass growth rate
$\beta_{\theta ,\lambda }$	the scattering phase function
${\left({\bar{\mu }}_{n}^{*}\right)}_{PAR}$	averaged coefficient of the phase function in the PAR range [rad]
${\left(\mu_{n}^{*}\right)}_{\lambda }$	coefficient of the phase function [rad]
Chl	chlorophyll
GRAS	generally recognized as safe
MC	Monte Carlo
OP	optical or radiative property
PAR	photosynthetic active radiation
PBR	photobioreactor
PBS	phycobilisome
PPF	photosynthetic photon flux [µmol s^−1^]
PPFD	photosynthetic photon flux density [µmol m^−2^ s^−1^]
PS	photosystem
REF	radiant energy field
RTE	radiative transfer equation
TPBR	tubular photobioreactor
