# Supplementary material for: The Assessment of the Real-Time Radiative Properties and Productivity of Limnospira platensis in Tubular Photobioreactors
Source: Life (Basel). 2022 Jul 8;12(7):1014. doi: 10.3390/life12071014 (PMC9320848; doi:10.3390/life12071014)
Supplement: Supplementary file 1 [file life-12-01014-s001.zip › life-1763665-supplementary.pdf]

SUPPLEMENTARY MATERIAL FOR:

‘The assessment of the real-time radiative properties and productivity of *Limnospira platensis* in Tubular Photobioreactors’

Manuel Vicente Ibañez [1,*], Rodrigo Jorge Leonardi [1], Felix Krujatz [2,3,4] and Josué Miguel Heinrich [1]

[1] Facultad de Bioquímica y Ciencias Biológicas (FBCB), Universidad Nacional del Litoral (UNL), Ciudad Universitaria (Paraje El Pozo), Santa Fe 3000, Argentina.

[2] Institute of Natural Materials Technology, TU Dresden, Bergstraße 120, 01069 Dresden, Germany.

[3] biotopa gGmbH—Center for Applied Aquaculture & Bioeconomy, Bautzner Landstraße 45, 01454 Radeberg, Germany.

[4] Faculty of Natural and Environmental Sciences, University of Applied Sciences Zittau/Görlitz, 02763 Zittau, Germany

* Correspondence: mibaniez@fbcb.unl.edu.ar (Manuel Vicente Ibañez).

(**a**)

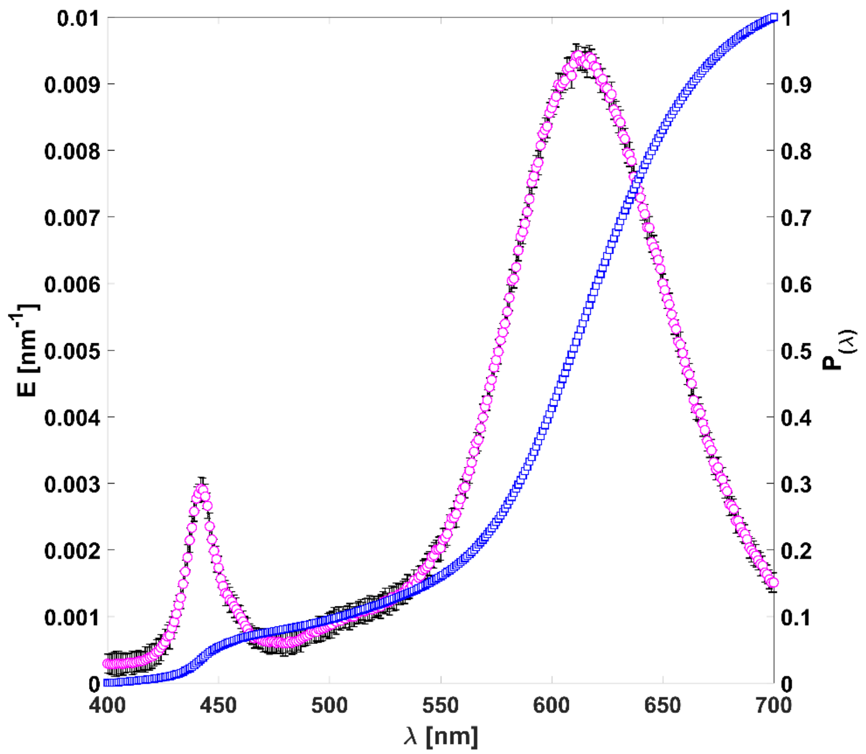

(**b**)

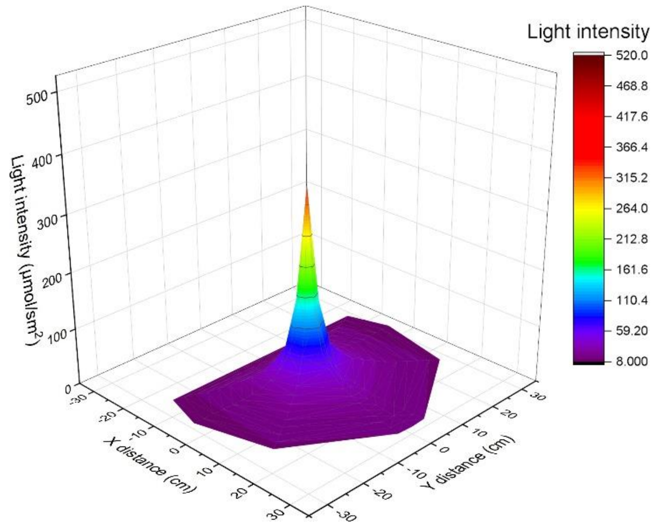

Figure S1. (**a**,**b**) The light source's spectral and directional distribution functions.

Table S1. The PAR average value of the coefficients generated by the optimisation algorithm, corresponding to the same series of absorption and scattering coefficients presented before.

	X [g L^{-1}]	0.111	0.152	0.245	0.330	0.541	0.611	0.674	0.715	0.747	0.763	0.764
$P(\mu_d) \geq 0.8$	$\left(\overline{\mu}_2^*\right)_{PAR}$	0.9979	0.9969	0.9950	0.9955	0.9969	0.9932	0.9944	0.9927	0.9953	0.9947	0.9941
$0.8 > P(\mu_d) \geq 0.6$	$\left(\overline{\mu}_3^*\right)_{PAR}$	0.9839	0.9919	0.9818	0.9842	0.9919	0.9861	0.9795	0.9911	0.9917	0.9916	0.9915
$0.6 > P(\mu_d) \geq 0.4$	$\left(\overline{\mu}_4^*\right)_{PAR}$	0.9223	0.9669	0.9451	0.9529	0.9491	0.9685	0.9353	0.9671	0.9781	0.9631	0.9541
$0.4 > P(\mu_d) \geq 0.2$	$\left(\overline{\mu}_5^*\right)_{PAR}$	0.7599	0.8079	0.7677	0.8607	0.7871	0.8714	0.7599	0.8171	0.8172	0.8121	0.7599
$0.2 > P(\mu_d)$	$\left(\overline{\mu}_6^*\right)_{PAR}$	0.5012	0.5508	0.6891	0.5607	0.5402	0.6851	0.6602	0.5307	0.4999	0.6710	0.6633

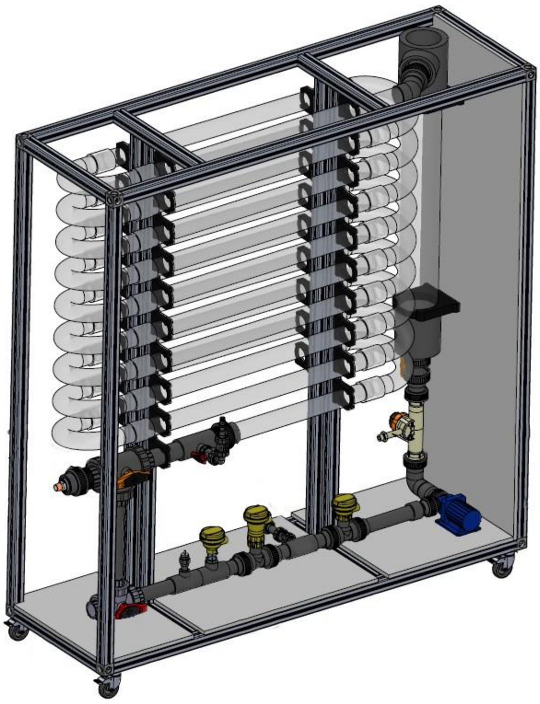

Figure S2. CAD rendering of 100L MINT PBR.

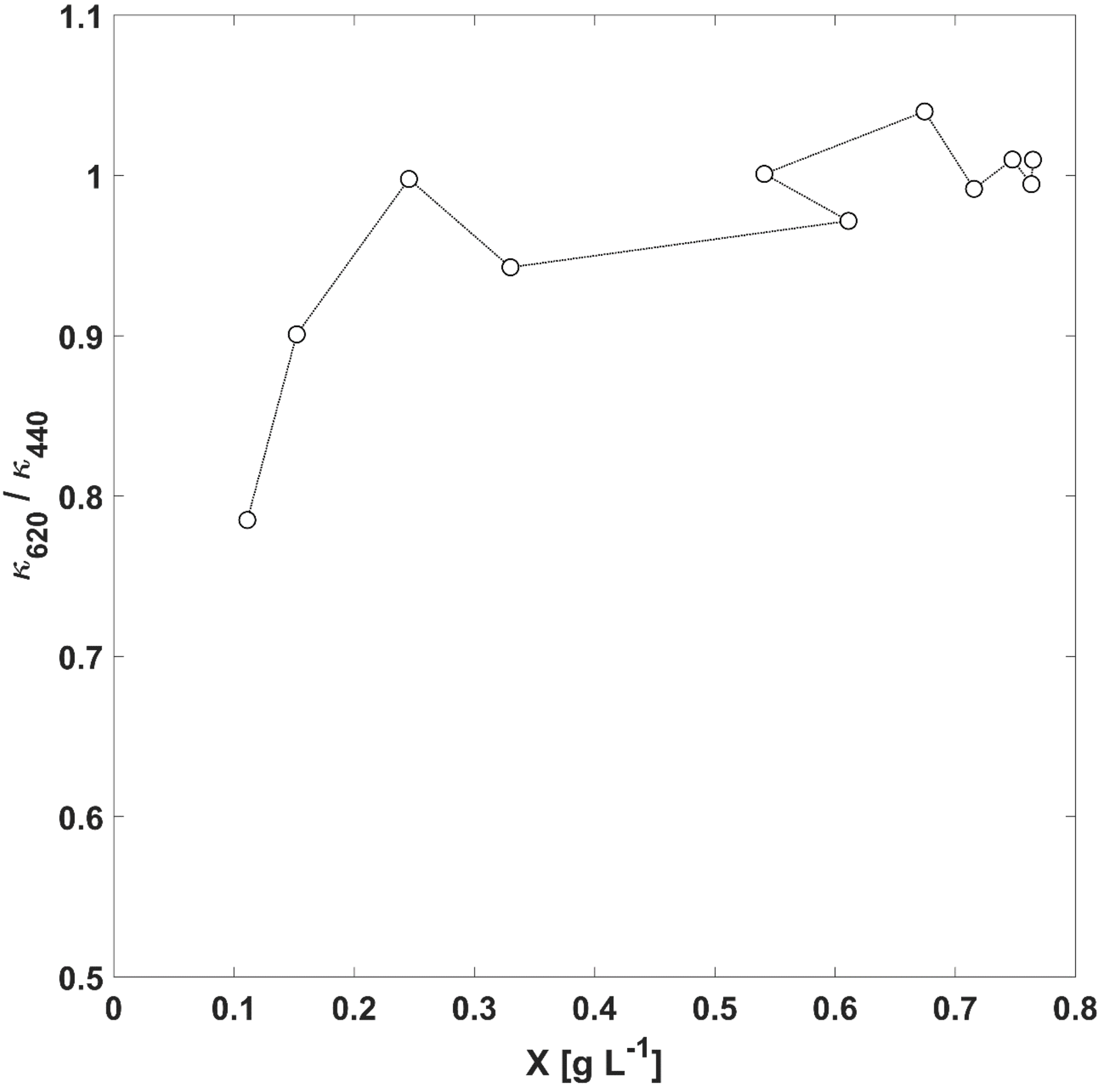

Figure S3. ratio of the κ_{620} and $\kappa_{440}$to observe the relative changes among the highest Chl-*a*-related and phycobiliprotein-related peaks.

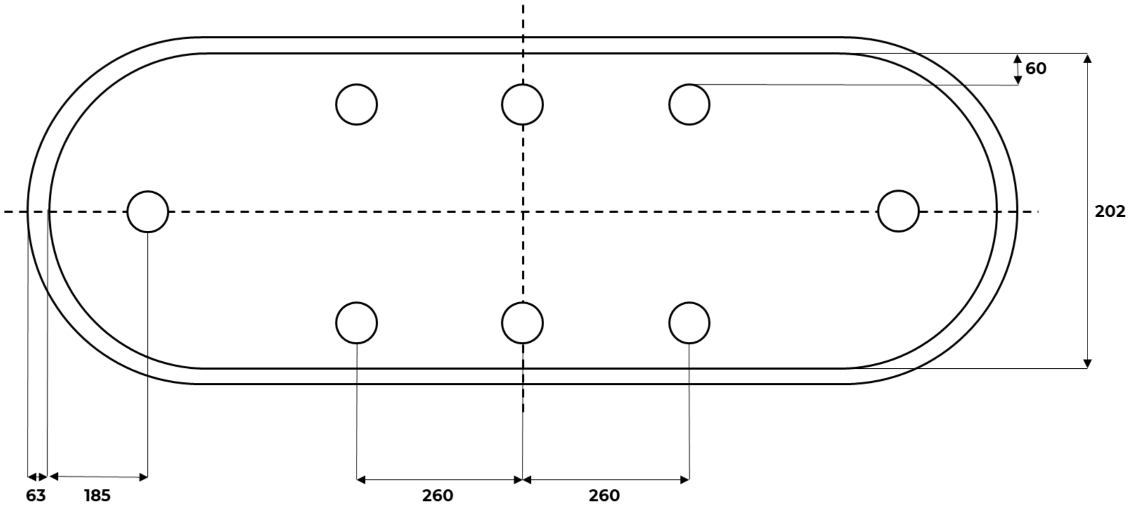

Figure S4. The schematic cross-section of the 100-L-MINT PBR. The figure is not a scaled representation but shows the position of the LED lamps from the PBR walls. All the measures are expressed in [mm].

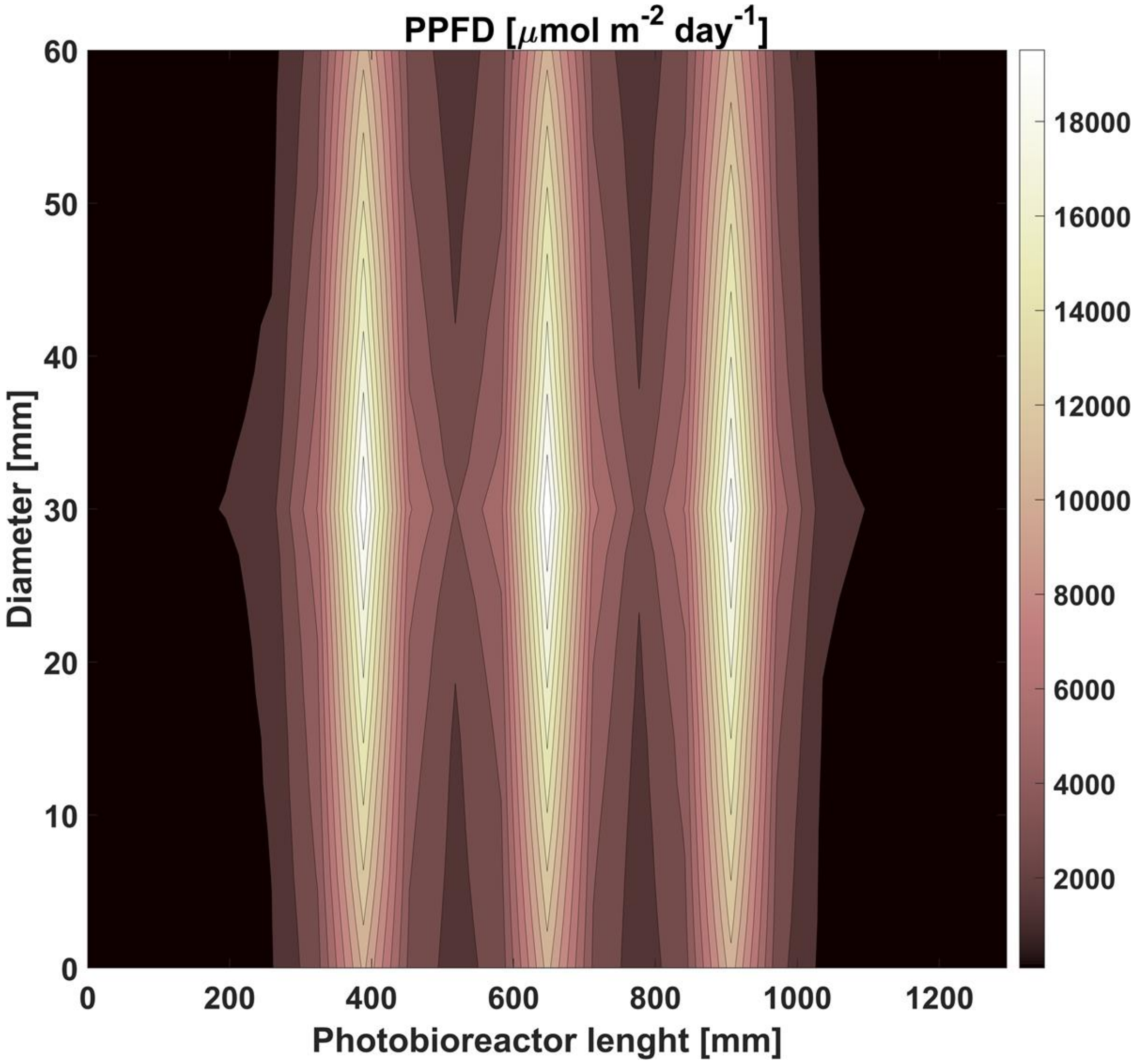

Figure S5. Photosynthetic photon flux density values over the photobioreactor surface facing the light source for the central tube of the 100-L-MINT PBR. This image is related to the previously presented figure (A4). The three LED lamps that face the reactor walls are related to the central position of the light dispersion presented in the PPFD of the present figure.
